# Leaf-rolling in maize crops: from leaf scoring to canopy-level measurements for phenotyping

**DOI:** 10.1093/jxb/ery071

**Published:** 2018-04-02

**Authors:** Frederic Baret, Simon Madec, Kamran Irfan, Jeremy Lopez, Alexis Comar, Matthieu Hemmerlé, Dan Dutartre, Sebastien Praud, Marie Helene Tixier

**Affiliations:** 1 INRA-EMMAH-CAPTE, Route de l’aerodrome, Avignon, France; 2 Biogemma, Route d’Ennezat, Chappes, France; 3 HIPHEN, Rue Charrue, Avignon, France

**Keywords:** Canopy structure, digital hemispherical photographs, FIPAR diurnal course, leaf rolling, maize, water stress

## Abstract

Leaf rolling in maize crops is one of the main plant reactions to water stress that can be visually scored in the field. However, leaf-scoring techniques do not meet the high-throughput requirements needed by breeders for efficient phenotyping. Consequently, this study investigated the relationship between leaf-rolling scores and changes in canopy structure that can be determined by high-throughput remote-sensing techniques. Experiments were conducted in 2015 and 2016 on maize genotypes subjected to water stress. Leaf-rolling was scored visually over the whole day around the flowering stage. Concurrent digital hemispherical photographs were taken to evaluate the impact of leaf-rolling on canopy structure using the computed fraction of intercepted diffuse photosynthetically active radiation, *FIPAR*_dif_. The results showed that leaves started to roll due to water stress around 09:00 h and leaf-rolling reached its maximum around 15:00 h (the photoperiod was about 05:00–20:00 h). In contrast, plants maintained under well-watered conditions did not show any significant rolling during the same day. A canopy-level index of rolling (*CLIR*) is proposed to quantify the diurnal changes in canopy structure induced by leaf-rolling. It normalizes for the differences in *FIPAR*_dif_ between genotypes observed in the early morning when leaves are unrolled, as well as for yearly effects linked to environmental conditions. Leaf-level rolling score was very strongly correlated with changes in canopy structure as described by the *CLIR* (*r*^2^=0.86, *n*=370). The daily time course of rolling was characterized using the amplitude of variation, and the rate and the timing of development computed at both the leaf and canopy levels. Results obtained from eight genotypes common between the two years of experiments showed that the amplitude of variation of the *CLIR* was the more repeatable trait (Spearman coefficient ρ=0.62) as compared to the rate (ρ=0.29) and the timing of development (ρ=0.33). The potential of these findings for the development of a high-throughput method for determining leaf-rolling based on aerial drone observations are considered.

## Teaser

The diurnal dynamics of leaf-rolling in maize genotypes as measured by visual scoring are strongly correlated with corresponding measurements made using digital hemispherical photography, leading to the potential for development of high-throughput techniques for phenotyping in the field.

## Introduction

Drought is recognized as one of the main factors limiting the production of maize crops (Farhangfar *et al.*, 2015). Plants have developed several mechanisms to mitigate the impact of environmental stresses, including ‘leaf-rolling’, in which the leaf lamina rolls transversally to the mid-rib under severe stress conditions. This mechanism results from a differential top–bottom elastic shrinkage in the leaf cross-section (Moulia, 2000). Leaf-rolling is thus related to the water potential in the leaf (Kadioglu *et al.*, 2012) and hence is a hydronastic response (Moulia, 2000). For maize, leaf-rolling is observed from leaf water potentials of –1 MPa and reaches its maximum around –2 MPa (Moulia, 1994). Rolling reaches its maximum close to solar noon during bright, sunny days (Kadioglu and Terzi, 2007) and the top leaves are generally most affected (Tatar *et al.*, 2010). Rolling occurs when the evaporative demand is no longer balanced by soil water extraction by the root system.

The leaf water potential is mostly controlled by the osmotic component through a range of biochemical pathways. Leaf-rolling has been related to the accumulation of phytohormones (Krishna, 2003; Takahashi and Kakehi, 2010; Talaat and Shawky, 2012), some of which control stress-responsive gene expression (Divi *et al.*, 2010). Some pronounced changes in concentrations of organic acids or ions such as K^+^ and Cl^−^ may also induce leaf-rolling, as demonstrated by Saglam *et al.* (2010). In addition to the biotic factors that had previously been reported, Kadioglu *et al.* (2012) demonstrated that herbivores, viruses, bacteria, and fungi may also induce leaf-rolling through other biochemical pathways.

Considering the leaf as a thin shell that conforms to the laws of mechanics, transversal leaf-rolling is coupled with longitudinal changes in leaf curvature (Hay *et al.*, 2000; Moulia, 2000). This makes the leaf stiffer, and it becomes more erect because the insertion angle that the leaf makes with the plant stem is usually closer to the vertical than is the average inclination of the whole leaf. The rolling of the leaf and its change in angle act to reduce the surface area that is exposed to sunlight. This potentially decreases both transpiration and photosynthesis at the canopy level (Abd Allah, 2009), although stomata are generally already closed under the stress conditions that prevail during leaf rolling, limiting the exchange of CO_2_ and water between the leaf and the atmosphere. Nevertheless, the boundary layer resistance of the rolled leaves is increased, limiting the leaf transpiration rate (O’Toole *et al.*, 1979). Another consequence of leaf-rolling is to generally re-orientate the leaf surfaces away from the direction of the sun (Smith, 1997). This reduces the of photon flux density per unit leaf area (Duncan, 1971), which limits leaf overheating and associated damage to the photosynthetic apparatus (Nar *et al.*, 2009; Sarieva *et al.*, 2010). Leaf re-orientation also affects the proportions of adaxial and abaxial surfaces exposed to the incoming light, and these have distinctive behaviors (Driscoll *et al.*, 2006; Soares *et al.*, 2008) with consequences on photosynthetic capacity and possible damage to the photosynthetic machinery. Leaf-rolling thus contributes to maintaining the internal plant water status (Subashri *et al.*, 2009). Over a longer time-scale, leaf-rolling may also be associated with a decrease in chlorophyll content due to the reduction of leaf area exposed to the sun, as proposed by Subashri *et al.* (2009); however, this could also mainly result from a direct effect of drought on chlorophyll content, as reported by Bolanos and Edmeades (1996).

Leaf-rolling as a consequence of water stress results from a combination of factors, including root development, the water extraction efficiency of the roots, adjustment of leaf area index, changes in canopy structure compared with non-stressed conditions, the leaf transpiration rate, and the sensitivity of the rolling mechanism to leaf water potential. Although leaf-rolling observed at the canopy scale appears to be a complex trait, it reveals key information on the strategy followed by plants subjected to stress conditions. The genetic diversity in maize shows a large range of drought tolerance that is exploited by plant breeders (Adebayo and Menkir, 2014). Leaf-rolling is one of a number of potential traits that may be used by breeders to evaluate drought resistance. It has already been associated with quantitative trait loci in rice (Price *et al.*, 2002) and durum wheat (Peleg *et al.*, 2009).

Several methods have been proposed to quantify leaf rolling. Sirault *et al.* (2015) evaluated the capacity of leaves to roll up under controlled conditions: leaf strips were immersed in polyethylene glycol solutions over a range of concentrations. After equilibrium was reached, the leaf cross-sections were imaged using micro-photographs, and the convex hull of the cross-sections were used to quantify leaf-rolling. Several methods have also been developed to evaluate the actual level of leaf-rolling under natural conditions. O’Toole *et al.* (1979) proposed the use of a template of schematic transversal leaf sections (from flat to completely rolled positions). This method was applied by Clarke (1986) to relate leaf-rolling to leaf water content. A similar scoring method based on the ratio of rolled to unrolled leaf width (Premachandra *et al.*, 1993) was used to relate leaf-rolling to drought tolerance (Saruhan *et al.*, 2012; Saglam *et al.*, 2014). All these methods of evaluation are mainly focused on leaf-level observations. Such low-throughput techniques are difficult to apply over large phenotyping experiments because of the highly dynamic nature of leaf-rolling. High-throughput methods are thus highly desired for field experiments.

The objective of this study was to evaluate the feasibility of a high-throughput method for monitoring the diurnal changes of canopy structure related to leaf-rolling. Ultimately, observations using multispectral cameras known to be sensitive to changes in canopy structure (Sankaran *et al.*, 2015) may be used to quantify leaf-rolling from aerial drone observations. However, the consistency between leaf-rolling at the leaf level and its impact on canopy structure first needs to be evaluated, and that was the focus of this study. In addition, because of the highly dynamic nature of leaf-rolling, this study also aimed at identifying pertinent and consistent traits that can be derived from the diurnal variation of leaf-rolling and that can be used to characterize the genotypic variability of plants subjected to water stress. Digital hemispherical photography (DHP) provides a very efficient way to document canopy structure through determination of the directional gap fraction (Jonckheere *et al.*, 2004; Weiss *et al.*, 2004; Lopez-Lozano *et al.*, 2007). The first section of this article details an experiment where a total of 30 and 20 genotypes, respectively, were studied in 2015 and 2016. Then, methods are described that were used to monitor the diurnal variation of leaf-rolling based on visual scoring and to monitor canopy structure as determined using DHPs. Based on the results, the consistency between leaf-level rolling and its impact on canopy structure is discussed, and several traits that characterize the diurnal dynamics of leaf-rolling are proposed as quantifiers of the genotypic reaction to water stress. Conclusions are finally drawn regarding the possible use of drone observations for high-throughput characterization of leaf-rolling.

## Materials and methods

### Field experiments

The field experiments were conducted near Nérac, France (44.17° N, 0.30° E) in 2015 and 2016. A total of 800 genotypes of maize (*Zea mays*) were grown in microplots consisting of two adjacent rows with 0.8 m spacing and 6 m long. The rows were oriented NW–SW. A subsample of 38 genotypes was selected for their large differences in canopy structure and susceptibility to leaf rolling. In 2015, 30 genotypes were maintained under severe water stress conditions (WS treatment). In 2016, 16 genotypes were maintained under similar severe water stress conditions, while four of them were also maintained under well-watered conditions (WW treatment). Eight genotypes were common to both years in the WS treatment while only two of them were also sampled in WW treatment in 2016 (Table 1). The soil water content at field capacity was 200 mm and the permanent wilting point (PWP) was 60 mm. In 2015, the soil moisture was below the PWP from 5th of July for the WS treatment, with only 25 mm of water storage remaining on 5th of August at the date of the leaf-rolling measurements. In 2016, the soil moisture was below PWP from the 8th of July, with 35 mm water storage remaining on 3rd of August at the date of the measurements. The water stress was therefore slightly greater in 2015 as compared to 2016.

**Table 1.** Distribution across experiments of the 38 genotypes used in 2015 and 2016 for water-stress (WS) and well-watered (WW) treatments

Genotype ID numbers	**WS 2015**	**WS 2016**	**WW 2016**	**Number of microplots**
16–37	X			22
10–15		X		6
2, 4, 5, 6, 7, 8	X	X		6
0, 1		X	X	4
3, 9	X	X	X	2
**Total**	**30**	**16**	**4**	**50**

**Fig. 1.**
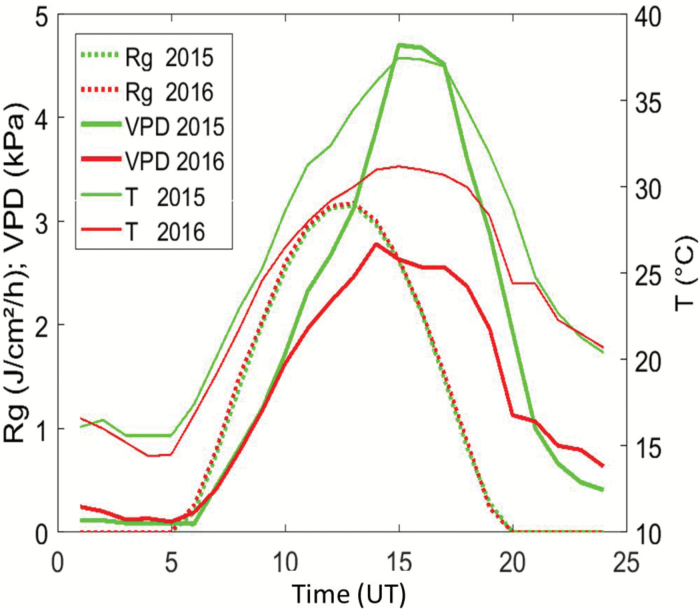
Diurnal variation of global radiation (Rg), temperature (T), and vapor pressure deficit (VPD) recorded on the days on which measurements were taken in 2015 and 2016. UT, universal time. (This figure is available in color at *JXB* online.)

The leaf-rolling measurements were performed approximately at the female flowering stage, which is known to be very sensitive to water stress. In both years, the measurements were conducted under very hot and sunny days with almost the same light conditions (Fig. 1). However, the 5th of August 2015 was more stressful as compared to the 3rd of August 2016, with higher temperatures and a much larger vapor pressure deficit (VPD), as calculated from air temperature and humidity after Monteith and Unsworth (2007).

### Visual scoring of leaf-rolling

Leaf-rolling was scored using a visual scale from 1 to 9: *Score* = 1 corresponded to no leaf-rolling (the cross-section of the leaf was almost flat), which was observed during the early morning or under no water-stress conditions; *Score* = 9 corresponded to maximum leaf-rolling, i.e. when the leaf cross-section was fully rolled. An average plant was scored by an observer positioned at one extremity of the microplot, and the leaves examined were those on the inside of the row, i.e. facing the adjacent row of the same genotype, so as to limit possible interactions with other microplots. All the microplots were scored by a single person for both experiments, to limit possible bias across the microplots, day and years. The scoring was repeated approximately every hour from 09:00 to 17:00 h (the photoperiod was about 05:00–20:00 h), and resulted in a total of 430 scores. It took about 15 min to score 30 plots. Note that the leaf scoring was done on only one of the three replicate microplots for each genotype.

### DHP measurements

Upward-looking digital hemispherical photographs (DHP) were taken using a sigma SD-14 camera equipped with a fisheye lens of 8 mm focal length. The camera was set on automatic exposure. The images were recorded in JPG format with 2640 × 1760 pixels. Images were repeated approximately every 1.25 h during the day from 09:00 to 17:00 h, resulting in seven time-steps. About 45 min were necessary to sample 30 microplots. The DHPs were taken in the same 30 (in 2015) and 20 (in 2016) microplots as the ones that were visually scored, i.e. with no replicates for each genotype.

**Fig. 2.**
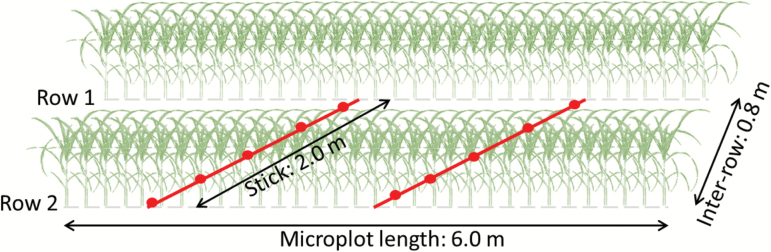
Location of digital hemispherical photography (DHP) measurements across the two rows of a microplot. A stick was placed in the positions shown and images were taken at the locations indicated by the circles. (This figure is available in color at *JXB* online.)

A total of 10 images were taken in each microplot at each time-step in the day to capture the spatial variability (Weiss *et al.*, 2004). The images were distributed over two diagonal segments placed between the two rows (Fig. 2). A 2-m long stick with marked positions was used to fix the precise positions of the camera for taking each image over a segment. The position of the stick in each microplot was kept the same across the repeated measurements during the day to provide a high degree of temporal consistency. The camera was always orientated in the same compass direction. Sampling a microplot with 10 images took about 1 min. Note that the hemispherical images partly included the neighboring microplots; however, the influence of this was limited because they were filtered to retain only pixels captured at zenith angles of less than 62.5° (see next section).

### Processing the DHP

**Fig. 3.**
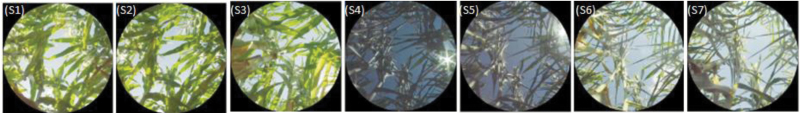
Example digital hemispherical photography (DHP) images taken at seven different time-points (from S1, morning, to S7, late afternoon) at the same location within a microplot. The changes of canopy structure from the morning to afternoon due to leaf rolling can clearly be seen. Some artifacts are apparent for S4 and S5 due to direct sunlight; however, these were fully accounted by the color classification that was used in image processing. (This figure is available in color at *JXB* online.)

The 3500 images (50 microplots with 10 images per microplot sampled seven times during the day) were processed using the CAN-EYE freeware (http://www6.paca.inra.fr/can-eye/CAN-EYE-Home/Welcome). CAN-EYE is a package of matlab functions developed to estimate canopy structure characteristics from RGB images (Demarez *et al.*, 2008). Classification between the sky and the vegetation elements was achieved based on the RGB color space with a predefined solution that was fine-tuned manually for each set of 10 images acquired over each microplot. Distinguishing between the two elements was relatively easy and non-ambiguous for all the seven time-steps considered (see Fig. 3). The calibrated values of the optical centre and projection function of the camera were used to compute the directional gap fraction *P*_o_(θ,φ) with 0°<θ<62.5° and 0°<φ<62.5°, where θ is the zenith angle (θ=0° corresponds to the nadir direction) and φ is the azimuth angle (φ=0° corresponds to the row direction). The zenith and azimuth directions were integrated into 2.5° steps. Angles from the zenith higher than 62.5° were discarded due to the large fraction of mixed pixels and the larger contributions of the neighbouring plots. Values from the 10 individual images were averaged to provide the microplot value of *P*_o_(θ,φ).

Given the assumed symmetry along and across the row directions, the directional gap fraction values were averaged to provide a representative quadrant, $\bar{P_{\text{o}}\left(\theta ,\;\varphi_{q}\right)}$ with 0°<φ_*q*_<π/2:

$$\bar{P_{\text{o}}\left(\theta ,\;\varphi_{\text{q}}\right)}=\frac{1}{4}\left(P_{\text{o}}\left(\theta ,\;\varphi_{\text{q}}\right)+P_{\text{o}}\left(\theta ,\;2\pi -\varphi_{q}\right)+P_{\text{o}}\left(\theta ,\;2\pi +\varphi_{q}\right)+P_{\text{o}}\left(\theta ,\;4\pi -\varphi_{q}\right)\right)$$ (1)

Several ‘segmental gap fractions’ were computed to investigate possible correlations with the leaf-rolling score. They corresponded to integration of *P*_o_(θ,φ) over a range of directions (θ,φ)to provide more stable directional gap fraction values: the hemisphere was divided into three different rings of 20° zenithal sectors: [0°<θ<20°], [20°<θ<40°], [40°<θ<60°]. In addition, each ring was divided into three azimuthal ranges: [0°<φ_q_<30°] corresponding to the row direction, [30°<φ_q_<60°] corresponding to a direction diagonal to the row direction, and [60°<φ_q_<90°] corresponding to the direction perpendicular to the row. Therefore, a total of nine segmental gap fractions were computed. In addition, the fraction of diffuse photosynthetically active radiation intercepted by the canopy, *FIPAR*_dif_, was also calculated:

$$FIPAR_{\text{dif}}=\;\frac{\sum_{0}^{\Pi /2}(1-P_{\text{o}}\left(\theta \right))cos\left(\theta \right)sin\left(\theta \right)}{\sum_{0}^{\Pi /2}cos\left(\theta \right)sin\left(\theta \right)}$$ (2)

Where *P*_o_(θ) was the azimuthally averaged value of *P*_o_(θ,φ). When evaluating *FIPAR*_dif_, values of *P*_o_(θ) for θ>62.5° were computed assuming a linear interpolation of the term [1–*P*_o_(θ)]cos(θ)sin(θ) between θ=62.5° and θ=π/2 for which [1–*P*_o_(π/2)]cos(π/2)sin(π/2) = 0.

## Results

### Diurnal variation of leaf-rolling visual scores

**Fig. 4.**
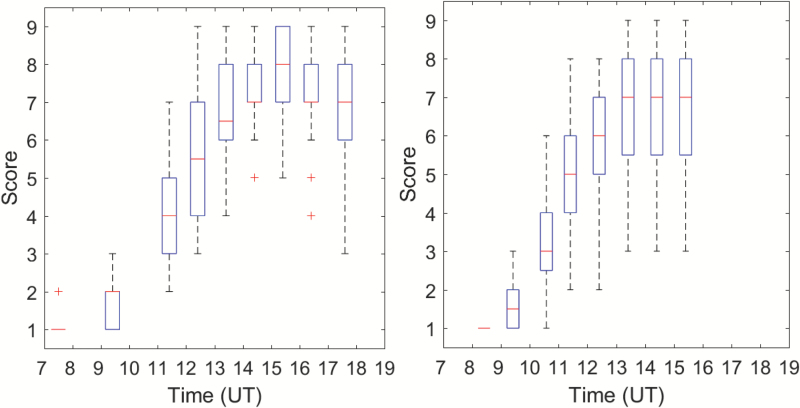
Diurnal pattern of the leaf-rolling scores for the water-stress treatment for 30 genotypes in 2015 (left) and 16 genotypes in 2016 (right). In the box-plots the line within the box is the median, the tops and bottoms of the box are the 75th and 25th percentiles, respectively, the whiskers extend to the most extreme data points that the algorithm considers not to be outliers, and outliers are plotted individually as ‘+’. (This figure is available in color at *JXB* online.)

In absence of water stress, leaves remained unrolled during the day, with *Score* = 1 for the four genotypes grown under well-watered conditions in 2016. Conversely, leaf rolling was observed for all the genotypes subjected to water stress in both 2015 and 2016. All the genotypes showed very similar diurnal patterns of leaf-rolling scores for both years. The score started from the minimum value, *Score* = 1, in the early morning (07:30 h) when no leaf rolling was observed (Fig. 4), although one cultivar already showed some leaf-rolling for the first scoring of the day in 2015. Leaves then rolled up progressively as the water stress experienced by the canopy increased as a function of the climatic demand, which was mainly controlled by the incoming radiation and the increasing vapor pressure deficit: at 09:00 h leaf-rolling was already observed in many of the genotypes in both years, when VPD≈1.5 kPa (Fig. 1). Maximum leaf-rolling was reached around 15:30 h, with some significant variation of the magnitude between genotypes (Fig. 4). This corresponded to the maximum value of the VPD during the day (Fig. 1). Finally, leaves started to unroll when the climatic demand decreased significantly. Variability at a given time between genotypes under water stress was maximum when the rate of increase of the leaf-rolling score was maximum, around 12:30 h (Fig. 4).

### Diurnal variation of the directional gap fraction

**Fig. 5.**
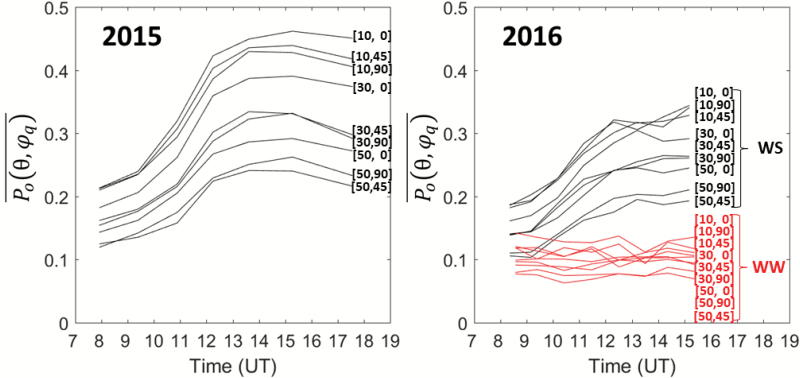
Diurnal pattern for nine segmental gap fractions, $\bar{P_{\text{o}}\left(\theta ,\;\varphi_{q}\right)}$ The directions (θ, φ_q_) are indicated at the end of each curve. $\bar{P_{\text{o}}\left(\theta ,\;\varphi_{q}\right)}$ was computed as the average for 30 genotypes in 2015 (water-stress treatment only), 16 genotypes in 2016 under water-stress (WS) and four genotypes in 2016 under well-watered (WW) conditions. (This figure is available in color at *JXB* online.)

Similar to the visual score, no clear diurnal variation of the gap fraction was observed for the well-watered genotypes (Fig. 5, ‘WW’ curves on the right). In contrast, the directional gap fraction increased during the day for the water-stressed genotypes in both 2015 and 2016 (Fig. 5, black curves). Very similar patterns were observed to those of the leaf-rolling scores, with a minimum value in the early morning, and a maximum value reached around 15:30 h, which corresponded to the maximum daily temperature and VPD values (Fig. 1). After this maximum value, leaves started to unroll at the end of the afternoon.

The same diurnal pattern was observed for all the directions considered, but with differences in minimum and maximum values (Fig. 5). As expected, higher gap-fraction values were observed close to the nadir and in directions parallel to the row. Conversely, the lowest gap fraction values were observed for directions perpendicular to the row and for the larger zenith angles. Whilst the largest diurnal variations were observed for directions near the nadir, a very strong consistency was observed for the diurnal patterns of all the directions considered (Fig. 5).

## Discussion

### Impact of leaf-level rolling on changes in canopy architecture

The leaf-level rolling as scored visually was tentatively related to the directional gap fractions as measured with the DHPs that examined the corresponding changes in canopy architecture. The fraction of intercepted radiation under diffuse light conditions (*FIPAR*_dif_) was selected to be used as a proxy for the canopy structure. This was supported by the very strong consistency between the gap fractions observed across the different directions (Fig. 5). The value of *FIPAR*_dif_ was computed from the directional integration of the gap fraction (eqn 2), which offered the advantage of smoothing out uncertainties associated with each individual segmental gap fraction measurement, thus providing more robust results.

**Fig. 6.**
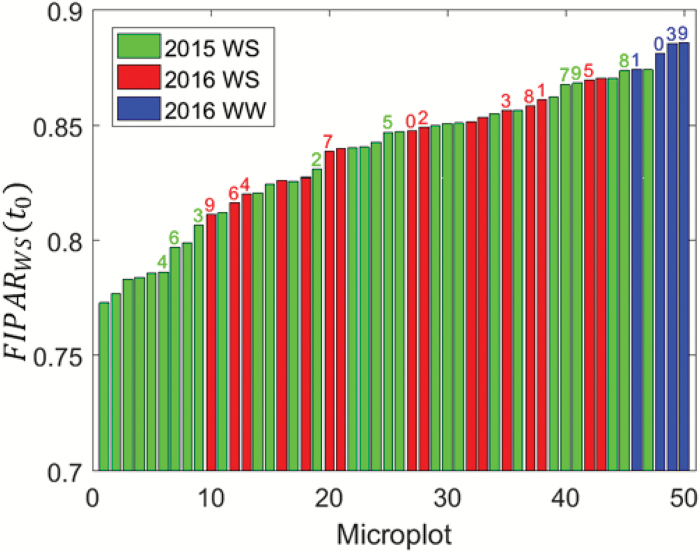
Distribution of the values for the fraction of intercepted diffuse radiation *FIPAR*_dif_(*t*_0_) observed in the early morning when leaves were in the unrolled state for all the 50 genotypes investigated. The values are sorted in ascending order. The years and treatments are indicated: WS, water stress; WW, well-watered. The identifiers of genotypes (Table 1) common between years and treatments are indicated above each bar. (This figure is available in color at *JXB* online.)

The visual score *Score*(*t*_0_), corresponding to the unrolled state of the leaf observed in the early morning (at time *t*_0_, i.e. before 9:00 h), was always set to *Score*(*t*_0_) = 1 for each genotype, except for one in 2015 as already noted (Fig. 4). However, at the canopy level, *FIPAR*_dif_(*t*_0_) values showed significant variability between genotypes when leaf rolling had not yet started (Fig. 6). This variability was explained by genotypic differences in the canopy architecture, such as differences in the leaf area index and/or plant morphology. Note that the well-watered (WW) treatment in 2016 showed higher *FIPAR*_dif_(*t*_0_) values as compared to the water-stress (WS) treatment since drought had already impacted leaf expansion during the weeks preceding the measurements. Similarly, the 2016 water stress was less severe than that of 2015, with generally larger *FIPAR*_dif_(*t*_0_) values in 2016. The plants thus developed differently depending on the environmental conditions they were subjected to, leading to obvious differences between years and treatments, and there was very little consistency between genotypes. Closer inspection of the distribution for the eight genotypes that were common between the two experiments in the WS treatment showed that the values of *FIPAR*_dif_(*t*_0_) observed in 2015 were not correlated with those observed in 2016 (*r*^2^=0.07, Spearman coefficient ρ=0.06).

**Fig. 7.**
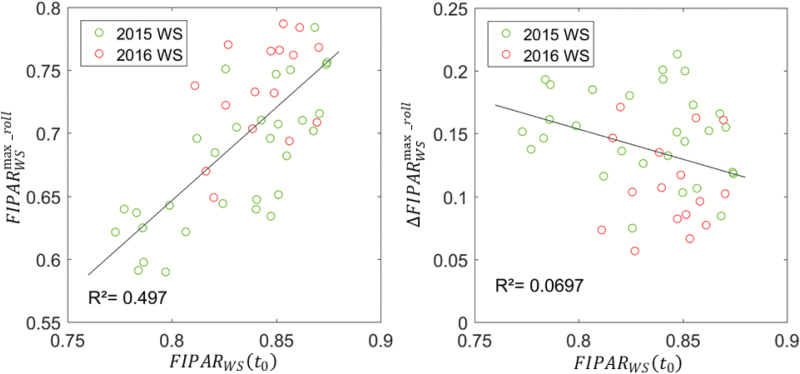
Left: the relationship between the fraction of intercepted diffuse radiation for unrolled leaves observed in the early morning, *FIPAR*_dif_(*t*_0_), and the values corresponding to the maximum leaf-rolling observed in the late afternoon, *FIPAR*_dif_^max_roll^. Right: the relationship between *FIPAR*_dif_(*t*_0_) and the difference in values observed between the early morning and the late afternoon, *ΔFIPAR*_dif_^max_roll^. The years and treatments are indicated: WS, water stress. The lines represent the linear best fit. (This figure is available in color at *JXB* online.)

The differences in *FIPAR*_dif_(*t*_0_) values in the early morning between genotypes and environmental conditions partly explained differences in the *FIPAR*_dif_ values observed during maximum leaf rolling, *FIPAR*_dif_^max_roll^, as shown in Fig. 7 (left panel; *r*^2^=0.50, *n*=46, correlation significant at α=5%). However, no significant (α>5%) correlation was observed between the difference *ΔFIPAR*_dif_^max_roll^ =*FIPAR*_dif_(*t*_0_) – *FIPAR*_dif_^max_roll^ and *FIPAR*_dif_(*t*_0_) (Fig. 7, right panel). A simple baseline normalization was therefore considered to limit the impact of the genotypic and environmental differences in the early morning: the *FIPAR*_dif_(*t*) values observed during the day at time *t* were subtracted from the unrolled *FIPAR*_dif_(*t*_0_) values observed in the early morning, i.e. *ΔFIPAR*_dif_(*t*)*=FIPAR*_dif_(*t*_0_) – *FIPAR*_dif_(*t*) 

**Fig. 8.**
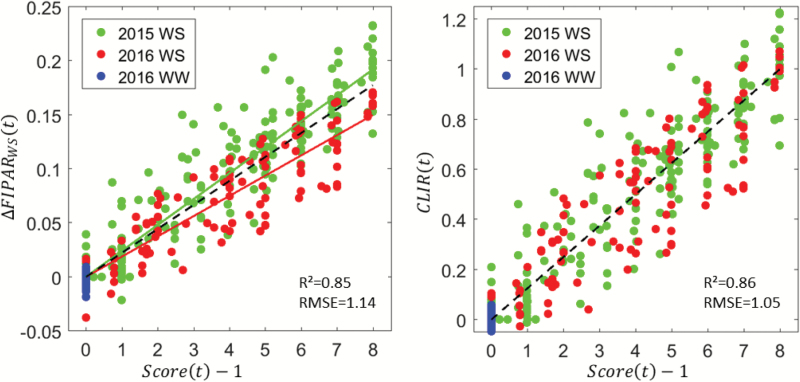
Left: the relationship between *ΔFIPAR*_dif_(*t*) and the leaf-rolling visual score [*Score*(*t*) – 1]. The solid lines correspond to the best-fit line verifying the constraint *ΔFIPAR*_dif_(*t*) = 0 when *Score*(*t*) = 1 (eqn 3) for the 2015 and 2016 water-stress (WS) treatments. The dashed line corresponds to the best-fit over all the 370 available data points including the well-watered (WW) treatment in 2016. Right: the relationship between the canopy-level index of rolling, *CLIR*(*t*) (eqn 4) and the leaf-rolling visual score [*Score*(*t*) – 1]. The dashed line corresponds to Equation 5.

The visual scores and the corresponding *FIPAR*_dif_(*t*) values need to be assigned to the same time during the day to establish a relationship between them. The visual scores were more frequently sampled and were therefore linearly interpolated to the time of the DHP measurements from which *FIPAR*_dif_(*t*) values were computed. The results (Fig. 8) showed that the constraint in the early morning between the leaf rolling score (*Score*(*t*_0_) = 1) and the *ΔFIPAR*_dif_ values (*ΔFIPAR*_dif_ (*t*_0_)=0) was well verified later in the day when no leaf rolling was experienced, such as for the well-watered treatment in 2016. The *FIPAR*_dif_ values were strongly and linearly related to the *Score* (Fig. 8). A simple linear model verifying the early morning constraint [*Score*(*t*_0_) = 1; *ΔFIPAR*_dif_(*t*_0_) = 0] was fitted to the available data:

$$\left[\text{Score}\left(t\right)\;–\text{ 1}\right]=Slope\times \Delta FIPAR_{\text{dif}}\left(t\right)$$ (3)

This model performed well for both years under water stress conditions (Table 2). For the well-watered treatment in 2016, the points were concentrated close to the unrolled-leaf situation with *Score*(*t*_0_) = 1 and *FIPAR*_dif_(*t*_0_) = 0. However, a small difference was observed between the two years, with 2016 showing lower sensitivity of the canopy structure (*ΔFIPAR*_dif_) to the level of leaf-rolling (*Score*). This may partly be attributed to the slightly smaller leaf development of the canopy in 2016, as illustrated in Fig. 5: when the canopy is less developed, limited absolute effects on canopy structure are expected. This effect can be accounted for by normalizing the values of *ΔFIPAR*_dif_ by the mean value observed for the maximum leaf rolling state, *ΔFIPAR*_dif_^max(year)^, leading to the proposal of a canopy level index for rolling, *CLIR*, with 0≤*CLIR*≤1:

**Table 2.** Characteristics of the models (eqns 3 and 4) used to relate the leaf-rolling visual scores to the canopy-level *FIPAR*_dif_ values

Model	Samples	Number of data points	Slope	*r* ^2^	RMSE
[*Score*(*t*) – 1] = Slope × *ΔFIPAR*_dif_(*t*)	2015 WS	210	41.64	0.857	1.05
	2016 WS	128	53.32	0.823	1.17
	All^(1)^	370	45.04	0.845	1.14
[*Score*(*t*) – 1] = 8 × *CLIR*(*t*)	All^(1)^	370	8	0.864	1.05
[max(*Score* – 1) = 8 × max(*CLIR*)	All^(1)^	50	8	0.771	1.15

RMSE is expressed in *Score* units. WS, water-stressed. ^(1)^ Includes the four 2016 well-watered (WW) microplots.

$$CLIR=\frac{\Delta FIPAR_{\text{dif}}(t)}{\Delta FIPAR_{\text{dif}}^{\text{max}(\text{year})}}$$ (4)

where *ΔFIPAR*_dif_^max(2015)^ = 0.19, *ΔFIPAR*_dif_^max(2016)^ = 0.16. *CLIR* is therefore related to the leaf-rolling score according to:

[Score(t)–1]=8×CLIR(t) (5)

The factor 8 ensures the normalization condition *CLIR* = 1 when *Score* = 9. The results showed a slight improvement in the performances of the regressions due to the enhanced consistency between years (Table 2).

### Comparisons between genotypes

Comparisons of the reaction to water stress between genotypes was undertaken by considering either the magnitude of the diurnal variation of leaf-rolling or the way it developed, i.e. the dynamics.

#### Magnitude of diurnal variation of leaf-rolling

**Fig. 9.**
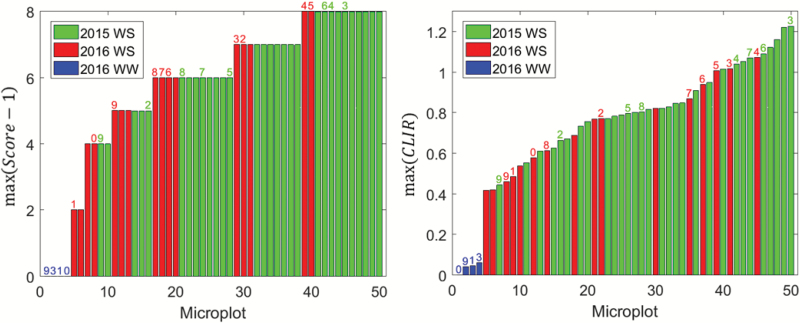
Left: distribution of the maximum value of the leaf-rolling visual score, *max*(*Score* – 1), observed for each microplot during the day. Right: distribution of the maximum value of the canopy-level index of rolling, *max*(*CLIR*), observed for each microplot during the day. The values are sorted in ascending order. The years and treatments are indicated: WS, water stress; WW, well-watered. The identifiers of genotypes (Table 1) common between years and treatments are indicated above each bar. (This figure is available in color at *JXB* online.)

**Fig. 10.**
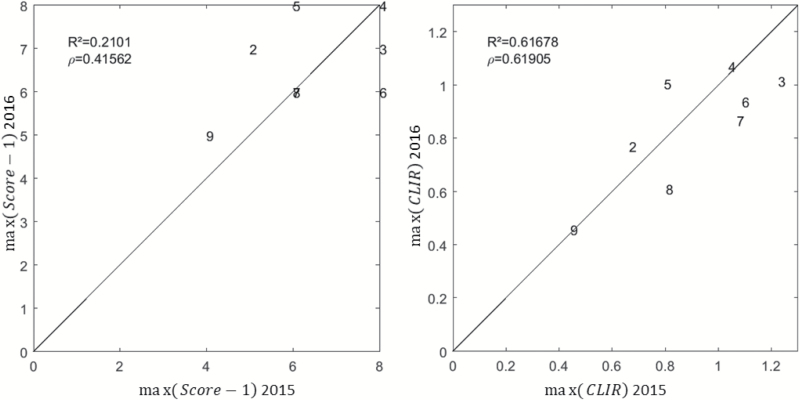
Left: comparison between the maximum value of the normalized scores, (*Score* – 1), observed in 2015 and 2016. Right: comparison between the maximum value of the canopy-level index of rolling, *CLIR*, observed in 2015 and 2016. The numbers correspond to the genotype identifier (Table 1). The 1:1 line is indicated. The Pearson (*r*^2^) and Spearman (ρ) coefficients are provided.

The magnitude of the variation of the leaf-rolling score over the day for each genotype was taken as the maximum value of (*Score* – 1). The well-watered treatment in 2016 consistently had a minimal magnitude because no leaf-rolling was experienced. Under water stress conditions, however, the genotypes showed notable differences (Fig. 9, left). About half the genotypes had a maximum variation in rolling that was lower than or equal to *Score* = 6, with a few of them only showing limited leaf-rolling at the end of the afternoon when it might be expected to be greater. There was generally less leaf-rolling in 2016, in agreement with our DHP measurements (Fig. 5). However, the scoring was not reporting this difference (Fig. 4), probably because the person was adapting his scoring scale, i.e. it was more subjective. The eight genotypes common between 2015 and 2016 showed relatively low levels of consistency in their ranking between the two years (Fig. 10, left). In contrast, a high degree of consistency was observed when using the *CLIR* to quantify leaf-rolling at the canopy level (Fig. 10, right). This demonstrated that the repeatability among years of the *CLIR* derived from DHPs was better than that of the visual scoring, which was mainly explained by the more objective values provided by the DHP measurements as compared to the visual scoring. Furthermore, the continuous values of *CLIR* (Fig. 9, right), as opposed to the discrete nature of the visual scoring, also contributed towards improve the accuracy of quantification of the leaf-rolling, and thus the repeatability among years. The magnitudes of rolling at the leaf and canopy levels were highly correlated, with *r*^2^=0.771 and RMSE=1.15 (Table 2).

#### Diurnal dynamics of leaf-rolling

**Fig. 11.**
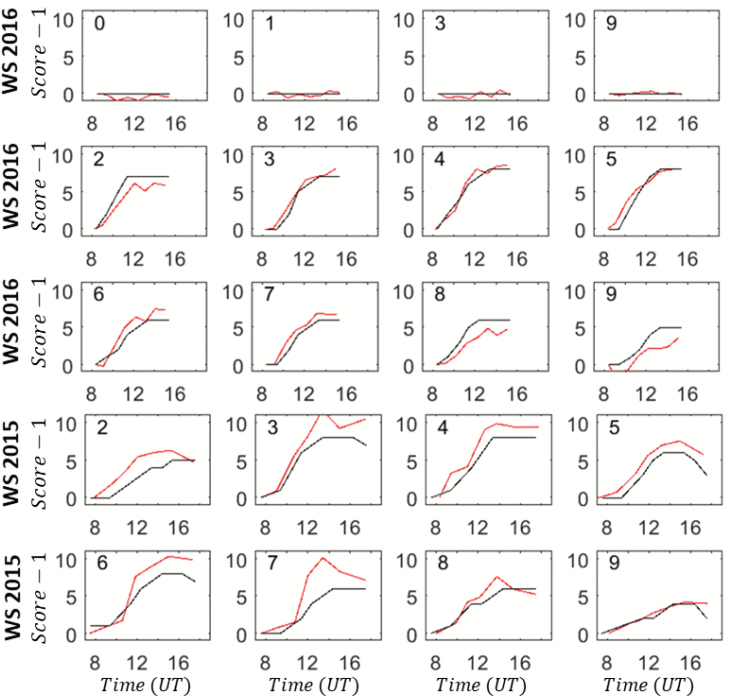
The diurnal dynamics of the leaf-rolling visual scores, (*Score* – 1), evaluated at the leaf level (black line), and the score estimated from eqn (5) (red line) from canopy-level digital hemispherical photography (DHP) measurements. The years and treatments are indicated: WS, water stress; WW, well-watered. WW 2016, four genotypes; WS 2016 and WS 2015, the same eight genotypes. The genotype identifier (Table 1) is indicated in each graph. (This figure is available in color at *JXB* online.)

Although the genotypic variability of the magnitude of rolling at the leaf and canopy levels was clearly one of the main features observed, we also examined other traits related to the diurnal dynamics. For this purpose, the leaf-level visual scores and the estimates from canopy-level *CLIR* values (eqns 4 and 5) were compared. The well-watered (WW) treatment showed no leaf rolling at all during the day together with no significant changes in the canopy structure (Fig. 11), which was consistent with observations of the gap fraction (Fig. 5). For the water stress (WS) treatment, Fig. 11 illustrates the very high consistency in the dynamics between the estimates of rolling at the leaf and canopy levels, confirming the previous results (Table 2 and Fig. 8). For each genotype, the dynamics of rolling at the leaf and canopy levels showed residual differences that were mainly in their maximum values, while their minimum values were generally very close together (Fig. 11). The values of the leaf- and canopy-level scores for rolling were further normalized by their observed minimum and maximum values between 08:00 to 17:00 h:

$$Score_{\text{norm}}=\frac{Score}{\max\left(Score\right)-\min\left(Score\right)}$$ (6)

**Fig. 12.**
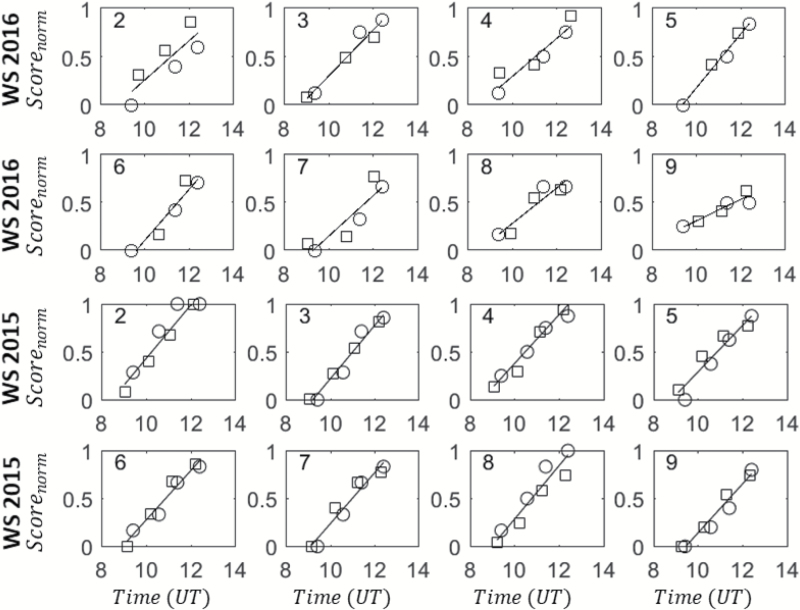
The dynamics of the normalized rolling score, (*Score*_norm_) evaluated at the leaf level (circles) and the canopy level (squares) using eqn (6) for the time interval 09:00 to 13:00 h (UT, universal time). The lines correspond to the best linear robust fit. The years and treatments are indicated: WS, water stress. The same eight genotypes were used throughout. The genotype identifier (Table 1) is indicated in each graph.

This normalization also allowed to separate the effect of the magnitude [max*(Score*) – min*(Score*)] from that of the dynamics. We then focused on the period of development of rapid leaf-rolling corresponding to the time interval from 09:00 to 13:00 h (Fig. 11). The very good consistency between the normalized score values, *Score*_norm_, at both the leaf and canopy levels is further illustrated in Fig. 12. The development of rolling appeared very linear with time at both leaf and canopy levels. A robust linear fit (Siegel, 1982) was thus applied to each microplot by using all the available data between 09:00 to 13:00 h, i.e. and merging the *Score*_norm_ derived at the leaf and canopy levels. The larger amount of data thus produced improved the accuracy with which the two main traits characterizing the dynamics were estimated, namely the slope (α) and the time at which half the maximum was achieved (*t*_max/2_).

$$Score_{\text{norm}}=0.\text{5}+\alpha \left(t–t_{\text{max}/\text{2}}\right)$$ (7)

**Fig. 13.**
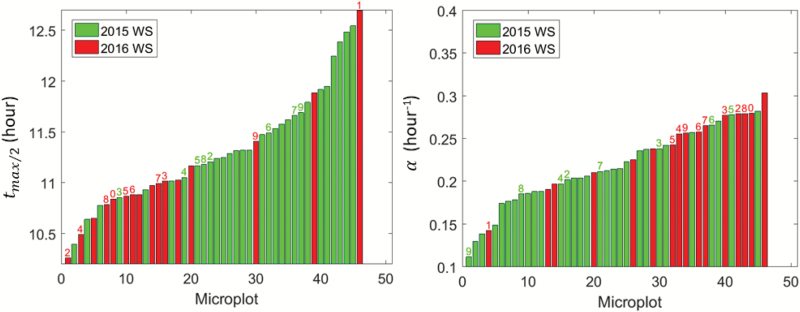
Patterns of distribution of the time (*t*_max/2_) when half the maximum rolling was observed (left), and the slope corresponding to the rate of change of rolling from minimum to maximum (α). The years and treatments are indicated: WS, water stress. The identifiers of genotypes (Table 1) common between years and treatments are indicated above each bar. (This figure is available in color at *JXB* online.)

The results showed that *t*_max/2_ varied strongly between genotypes and years, with 11.15< *t*_max/2_<12.45 (Fig. 13, left). Earlier values for *t*_max/2_ were observed in 2016 as compared to 2015. We examined the consistency between the eight common genotypes across the two years, in a similar way to what was done for the magnitude (Fig. 10). However, relatively poor consistency was observed (ρ=0.33, *r*^2^=0.49). The slope (α) also showed a large variability between genotypes and years (0.1<α<0.3 (Fig. 13, right). The consistency between the eight common genotypes across the two years for α values was even poorer (ρ=0.29, *r*^2^=0.21) than that of *t*_max/2_. In contrast to *t*_max/2_, larger values of α were generally observed in 2016 (Fig. 13). A negative correlation linked *t*_max/2_ and α (*r*^2^=0.42): since rolling started to develop at approximately the same time (between 08:15 to 09:30 h, Fig. 11), the value of the half-magnitude was reached earlier for larger slopes.

## Conclusions

This study focused on leaf-rolling in maize crops subjected to water stress. For both years of experiments, the soil moisture was below the permanent wilting point and high VPD values were observed during the day when the measurements were taken around the time of the female flowering stage. As a consequence, leaf-rolling started to become significant after 09:00 h, when VPD≈1.5 kPa, and reached its maximum value around 15:30 h, close to the maximum VPD value of the day. Differences between genotypes and years were mostly related to the maximum leaf-rolling score that was observed. Lower score values were recorded in 2016 and this was related to the lower water stress that was experienced (more water in the soil, lower VPD values) as compared to 2015. However, the eight genotypes sampled across both years showed a relatively good consistency of their ranking for the maximum score values observed (ρ=0.41), which indicates some useful degree of heritability. Further measurements conducted across the whole array of microplots of the two experiments would be needed to confirm the level of heritability of the leaf-rolling. However, scoring from visual inspection of hundreds of microplots within a limited time period is not practically feasible because of the highly dynamic character of leaf-rolling. Alternative high-throughput phenotyping methods are thus highly desired. Hence this study concurrently investigated the impact of leaf-rolling on canopy structure, which can be assessed using high-throughput remote sensing techniques. However, before developing an operational system, the main objective of this study was to conduct a comparison between leaf-level and canopy-level rolling features.

Changes in canopy structure due to leaf-rolling were recorded using DHP measurements. Detailed examination of the relationship between changes in gap fractions derived from DHPs and leaf-level visual scoring of rolling showed strong correlations for all the directions considered. For this reason, the fraction of intercepted diffuse photosynthetically active radiation, *FIPAR*_dif_, was selected as a good proxy of the changes in canopy structure induced by leaf-rolling: it retains the main changes while smoothing out uncertainties associated with limited directional sampling of the gap fraction. To compensate for the possibility of inherent differences between experimental conditions and genotypes, values of *FIPAR*_dif_ in the early morning when no leaf-rolling is expected were subtracted from the values measured during the day, to get *ΔFIPAR*_dif_. However, the values of *ΔFIPAR*_dif_ observed around 15:00 h when the maximum leaf-rolling is expected may differ from year to year depending on the experimental conditions. This effect was therefore accounted for by further normalizing *ΔFIPAR*_dif_ using the average of the maximum values that were observed during the day across all the microplot measurements. This resulted in the canopy-level index for rolling, *CLIR*. The agreement between the rolling of the leaf cross-section and the opening of the canopy as quantified by the *CLIR* appeared to be very strong and relatively stable across genotypes and across the two years of experiments. Furthermore, an improved degree of consistency (ρ=0.62) was observed between the ranking of the maximum *CLIR* values of 2015 and 2016 among the eight genotypes common for both years.

We also examined other features associated with the dynamics of leaf-rolling. The time at which half the maximum magnitude of the leaf-rolling effect was reached (*t*_max/2_) was not easy to estimate and showed significant variability between genotypes and years. Earlier values of *t*_max/2_ were generally observed in 2016 even though the water stress was less severe as compared to that experienced in 2015. The consistency of the ranking between the eight common genotypes across the two years was poor (ρ =0.33), making *t*_max/2_ a difficult trait to use for breeding. The rate of development of leaf-rolling as determined at the leaf (*Score*) and canopy (*CLIR*) levels showed little difference between years, especially for *CLIR*. Although the genotypic variability was significant, the consistency of the ranking between the common genotypes for the two years was poor, both for the rate of change of the score (ρ=0.21) and for *CLIR* (ρ=0.10). As a result, the main trait that appeared to be related to genotype was the magnitude of the rolling that occurred between the early morning and the time of maximum rolling. The results were, however, only supported by a limited dataset: only two years of experiments with a strong water stress treatment, with observations conducted at the same growth stage (flowering), based on few genotypes and with no replicates. Our findings therefore need further verification on a larger scale in order to quantify the heritability and to determine possible association with markers in the genome. A high-throughput phenotyping method therefore needs to be developed to estimate leaf rolling from canopy-level measurements. The use of unmanned aerial vehicle (UAVs) equipped with multispectral cameras would provide a very efficient way to cover a large experiment within a limited time period: the canopy reflectance measured over a few bands can be used to estimate FIPAR, as demonstrated by several studies (Asrar *et al.*, 1989; Baret *et al.*, 2013). A minimum of two flights would be necessary: one in the early morning to record the unrolled state, and one in mid-afternoon to quantify the canopy structure when leaf-rolling is at its maximum. The measurements should be conducted during periods when water stress is already well expressed, and on days with a high VPD values to maximize plant reactions. The relationship between the data captured by the multispectral camera and the leaf-rolling state could be simply determined using empirical transfer functions. A representative sample of ground measurements would therefore need to be collected concurrently with the flights to calibrate the transfer functions. The proposed *FIPAR*_dif_ variable, derived from DHPs according to the methodology presented in this study, would provide an efficient solution.

Although leaf-rolling can be quantified both at the leaf and the canopy levels, as demonstrated in this study, the effects of differences between genotypes in terms of physiological response to water stress remain to be determined. Differences between genotypes may relate to variations in soil moisture due to differences in water consumption or rooting-system development and efficiency. They may also relate to the regulation of stomatal closure, as well as variations resulting from differences in morphology that affect the relationship between rolling at the leaf level and leaf water potential. Furthermore, while this study showed that the leaf-rolling induced canopy-level changes that were relatively stable across genotypes, possible residual genotypic effects may complicate the interpretation. Detailed studies are therefore required to better understand the mechanisms that influence leaf-rolling under water stress conditions.
